# Diagnostic progression to hoarding disorder: the longitudinal course of hoarding behavior

**DOI:** 10.1186/s12888-026-08055-4

**Published:** 2026-04-16

**Authors:** Mehmet Akif Akıncı, Bahadır Turan, Esen Yıldırım Demirdöğen, Abdullah Bozkurt, Mehmet Ali Donbaloğlu, Gülsüm Tuğba Korkmaz Ürük, İbrahim Selçuk Esin, Onur Burak Dursun

**Affiliations:** 1 Department of Child and Adolescent Psychiatry, Atatürk University Faculty of Medicine, Erzurum, Türkiye; 2 Department of Child and Adolescent Psychiatry, Karadeniz Technical UniversityFaculty of Medicine, Trabzon, Türkiye; 3 Cambridgeshire and Peterborough Foundation Trust, Cambridge, UK; 4 Department of Child and Adolescent Psychiatry, Erzurum City Hospital, Erzurum, Türkiye; 5 Department of Child and Adolescent Psychiatry, Trabzon University Faculty of Medicine, Trabzon, Türkiye

**Keywords:** Hoarding disorder, Disease progression, Risk factors, Adolescent, Child, Longitudinal studies

## Abstract

**Background:**

The longitudinal course of hoarding behavior (HB) in children and adolescents, as well as the predictors of its outcome, remain unknown. This prospective longitudinal study aims to evaluate the four-year course of HB and identify predictors of progression to hoarding disorder (HD) and the persistence of hoarding symptoms.

**Methods:**

Children and adolescents with HB underwent a clinical assessment after a four-year follow-up period. The assessment included a one-on-one psychiatric interview, an HD clinical interview based on DSM-5 diagnostic criteria, Schedule for Affective Disorders and Schizophrenia for School-Age Children-Present and Lifetime version (K-SADS-PL), a semi-structured diagnostic interview for comorbid diagnoses, and the Children’s Saving Inventory (CSI) to assess hoarding symptom severity.

**Results:**

Over the 4-year follow-up period, 42.2% (*n* = 19) of participants progressed to HD, 20% (*n* = 9) persisted in HB, and 37.8% (*n* = 17) no longer displayed hoarding symptoms. Baseline hoarding symptom severity significantly predicted progression to HD and was also associated with persistence of hoarding symptoms. In exploratory models, current psychiatric comorbidity and female gender were associated with persistence but not progression.

**Conclusions:**

These findings provide precious insights into the longitudinal course of HB and predictors. The results also suggest the importance of early identification and monitoring of HB.

**Supplementary Information:**

The online version contains supplementary material available at 10.1186/s12888-026-08055-4.

## Introduction

Hoarding behavior (HB) is characterized by a persistent need to save items, regardless of their actual value, and significant difficulty in discarding personal possessions [1]. Traditionally, HB has been considered a symptom cluster linked to obsessive-compulsive disorder (OCD). However, studies on the nosology of hoarding have demonstrated that this classification may not be substantiated [2]. In light of growing evidence, hoarding was recognized as a distinct disorder—hoarding disorder (HD)—in the Diagnostic and Statistical Manual of Mental Disorders, Fifth Edition (DSM-5) [3]. The prevalence of HD among children and adolescents is estimated to be approximately 0.98% [4], with a gradual increase in prevalence observed with increasing age [5]. Retrospective studies of adults with HD report that the initial symptoms manifest during childhood and adolescence and follow a chronic course [6]. Given the recent classification of HD as a distinct disorder, little is known about its longitudinal course and outcomes, particularly in children and adolescents.

A review of the literature reveals that studies on hoarding in children and adolescents have predominantly focused on prevalence [4, 7], and correlates between hoarding and other psychiatric disorders, including OCD [8], attention deficit/hyperactivity disorder (ADHD) [7], and anxiety disorders [9]. However, since a significant proportion of these studies did not incorporate clinical interviews, it remains uncertain whether the diagnostic criteria for HD were fully met. Furthermore, only a handful of studies have evaluated hoarding with DSM-5-based clinical interviews [4, 10]. On the other hand, there is a lack of research on follow-up studies of hoarding in childhood and adolescence. To date, only one study has longitudinally evaluated adolescents with hoarding symptoms after a median of three years [10]. The study by Ivanov et al. examined the developmental origins of HD and reported that a non-negligible proportion of young people with hoarding symptoms exhibited significant hoarding symptoms at follow-up. However, that investigation did not evaluate predictors of the persistence of hoarding symptoms.

No prior study has identified predictors of persistence of hoarding symptoms or, more specifically, diagnostic progression to HD. This constitutes a significant research gap, as it is possible to clinically evaluate predictive variables associated with progression to disorder. Although few studies in adult samples suggest that HB is a precursor and key component of HD [11, 12], comparable longitudinal data in children and adolescents are lacking. Our previous cross-sectional findings indicated that children and adolescents with HB may constitute a high-risk group for HD [4]; however, cross-sectional designs cannot determine temporal progression or clarify which individuals are more likely to experience persistence or diagnostic progression. There is a clear need to identify predictors associated with progression to HD in children and adolescents with HB and to conduct follow-up studies with clinical interviews to clarify the longitudinal course and outcomes of HB. Clarifying the course and identifying predictors will also enable the development of prevention and early intervention programs.

To this end, we designed a prospective longitudinal study including a DSM-5-based HD clinical interview. In our previous study evaluating the prevalence and correlates of HB and HD [4], we followed-up with children and adolescents with HB who had no psychiatric diagnosis or symptoms. First, we aimed to evaluate the four-year longitudinal course of HB. Since this was the first follow-up study of HB, we could not fully predict the course, but we hypothesized that three different scenarios could occur: HB might progress to HD, persist or disappear. Second, we evaluated predictors of progression to HD and the persistence of hoarding symptoms. Based on the results of our previous study, we predicted that female gender and the presence of any comorbid psychiatric disorder would be predictors of the outcome.

## Method

### Participants

The present study sample consists of children and adolescents with HB from an epidemiological study conducted in 2018 [4]. In the previous study, participants were between 10 and 14 years old. More information about the sample can be found in the original prevalence article. In that study, individuals who met DSM-5 diagnostic criteria A, B, E, and F for HD (i.e., those who did not have sufficient functional impairment to be diagnosed with hoarding disorder) were included in the present study. The previous study, which provided the data source for this research, identified HB in 61 children and adolescents. Twelve participants with psychiatric comorbidities and subthreshold psychiatric symptoms at that time were excluded. The target population of the present study consisted of 49 participants with HB and no psychiatric symptoms.

**Fig. 1:**
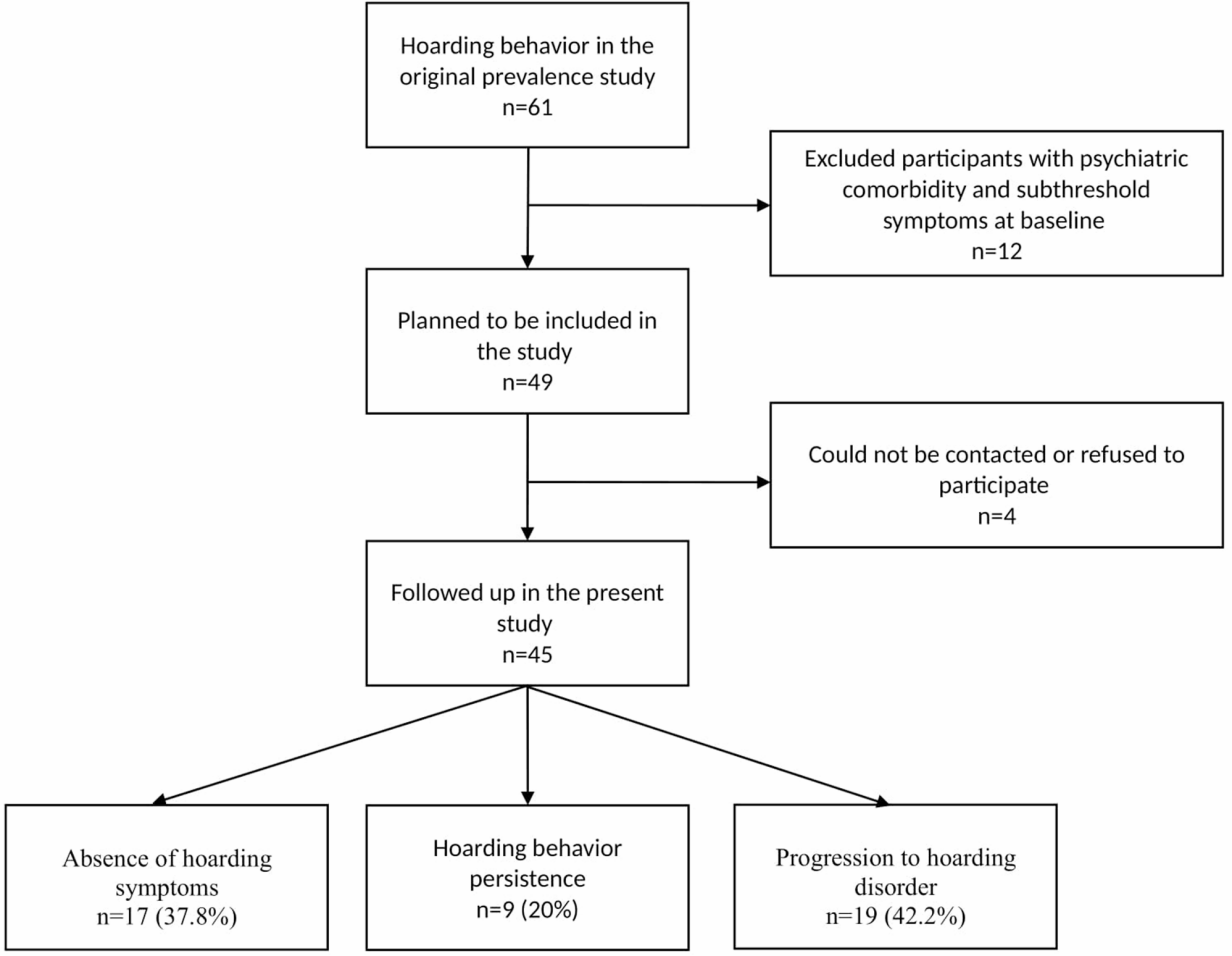
Flow chart of the study

All participants were invited to participate in a face-to-face psychiatric interview four years after the initial assessment using the contact information provided during the previous study. Four participants who could not be contacted or refused to participate were excluded. In total, 45 adolescents (15 males and 30 females; age range 14–18 years) and their parents agreed to participate in the follow-up interviews and provided written informed consent for the study. The study flow is illustrated in Fig. 1.

### Procedure

Ethical approval was obtained from the Ethics Committee of the Faculty of Medicine. This study was conducted as a single-center longitudinal follow-up study at the Child and Adolescent Psychiatry Department of the Atatürk University Faculty of Medicine, Erzurum, Türkiye, in 2022. This research serves as a follow-up to the epidemiological study that examined the prevalence of hoarding disorder and hoarding behavior [4].

After four-year follow-up period, adolescents and their parents were invited for a second interview. Follow-up assessments were conducted in person through face-to-face psychiatric interviews with adolescents and their parents. Each assessment lasted an average of 90 min. Those who attended were administered the following assessments:

- One-on-one psychiatric interview
- HD and HB clinical interview (to assess DSM-5 diagnostic criteria)
- Schedule for Affective Disorders and Schizophrenia for School-Age Children–Present and Lifetime Version (K-SADS-PL) (a semi-structured diagnostic interview for comorbid diagnoses)
- Children’s Saving Inventory (CSI) (to measure hoarding symptom severity)

All assessments were conducted by two experienced child and adolescent psychiatrists. To ensure diagnostic consistency, inter-rater reliability was assessed in a randomly selected group of patients who had also visited the clinic. The agreement between raters was high (Kappa value = 0.88), indicating excellent reliability. Final diagnostic decisions were reached through consensus meetings. Possible predictive demographic and clinical characteristics associated with diagnostic progression to HD were evaluated in psychiatric interview.

During the four-year follow-up period, none of the participants received psychotherapy or pharmacological treatment specifically targeting hoarding symptoms. However, 12 of the 45 participants had previously received psychiatric treatment for attention deficit/hyperactivity disorder (ADHD), anxiety disorders, depressive disorders or obsessive-compulsive disorder (OCD). Pharmacotherapy primarily consisted of standard evidence-based medications for these conditions (e.g., stimulants, selective serotonin reuptake inhibitors), and cognitive behavioral therapy (CBT) was provided when clinically indicated for the respective diagnoses.

In the HD and HB clinical interview, DSM-5 diagnostic criteria were assessed. Given the developmental context of adolescence and the well-documented influence of parental control over the living environment, Criterion C was evaluated with developmental flexibility rather than strict reliance on observable clutter alone. In line with prior pediatric hoarding research, parental intervention of the home environment was explicitly considered during diagnostic assessment. Criterion C was considered fulfilled when either (1) clutter was directly observable, or (2) parents reported active restriction of accumulation behaviors that would otherwise result in significant clutter in the absence of intervention. To support this determination, parents were systematically asked how the adolescent’s living space would appear if no parental intervention occurred. In fact, the findings from our previous study suggest that the distinction between HD and HB is not solely based on criterion C endorsement. Our previous study emphasized that one of the most significant clinical differences between these conditions is the endorsement or non-endorsement of criterion D. Based on our previous study results:

- Participants meeting criteria A and B, but not C and D, were classified as HB.
- Participants meeting criteria A and B, as well as C (directly or indirectly) and D, were classified as HD.

The emphasis on diagnostic criterion D stems from our strict adherence to the DSM criterion of functional impairment. Participants with HD and HB also met criteria E and F.

At the end of the four-year follow-up period, participants with a prior diagnosis of HB were classified into three groups:

- Progression to HD
- Persistence of HB
- Absence of hoarding symptoms

## Measures

### Sociodemographic data form

This form queried the demographic data of the children and their parents. Information on family psychiatric history, family income and family type were obtained from parental report. Family income level categorized according to locally applicable socioeconomic standards (e.g., low/middle/high) based on monthly household income. Stressful life events during the four-year follow-up period were assessed retrospectively using a checklist. Parents (and adolescents when appropriate) were asked whether each of the following events had occurred at any point during the preceding four years: parental divorce, pregnancy in the family, excessive family debt, relocation to a new place (town/city/home), a marked decrease in family income, presence of substance or alcohol problems in the family, death of a close family friend, job loss of one parent, legal problems experienced by a parent, and loss of a close family member. History of any psychiatric treatment during the four-year follow-up period was assessed through review of available medical records and corroborated by parental and adolescent report. Treatment exposure was included any pharmacological treatment or psychotherapy provided for psychiatric conditions during the follow-up interval.

### Schedule for affective disorders and schizophrenia for school-age children–present and lifetime version (K-SADS-PL)

This schedule was developed by Kaufman et al. (1997) [13] as a semi-structured interview tool used to assess past and present symptoms in children aged 6 to 18 years. The interview schedule was later revised to comply with DSM-5 diagnostic criteria. In the first part of the interview schedule, basic information about the sociodemographic characteristics, complaints, developmental history, and general functioning of the child and family are asked in an unstructured interview. The second part includes screening questions assessing more than 200 specific symptoms, past and present (in the last two months). The third part consists of assessment and observation results to confirm DSM-5 diagnoses. At the end of the interview, the clinician’s observations and the information received are evaluated and scored together. The Turkish validity and reliability study was conducted by Ünal et al. (2018) [14].

### Children’s saving inventory (CSI)

The CSI is a parent-rated scale developed to measure the severity of hoarding symptoms in children and adolescents [15]. Each item score on the scale ranges from 0 to 4, with higher scores indicating more severe hoarding symptoms. Although the original scale consisted of 23 items, the developers removed three items from the scale due to low correlations and factor loadings. The Turkish validity and reliability of the scale was conducted by Akıncı et al. (2023) [16]. Three items were removed from the Turkish version of the scale due to low item-total correlations and factor loadings. Overall, the 20-item CSI Turkish version has been demonstrated to be a valid and reliable scale that can be used in children and adolescents in a clinical sample.

### Statistical analysis

All statistical analyses were primarily conducted using SPSS version 26.0 (IBM Corp., Armonk, NY). The normality of continuous variables was assessed using the Shapiro–Wilk test. Independent samples t-tests were used for normally distributed variables, whereas the Mann–Whitney U test was applied when distributional assumptions were not met. Comparisons between groups were made using Pearson’s chi-squared test or Fisher’s exact test for categorical variables. First, the four-year course of HB was described. Second, we compared (1) participants who progressed to HD with those who did not progress (HB persistence and no diagnose), and (2) participants with persistence of hoarding symptoms (progression to HD and HB persistence) with those who were not diagnosed. Third, two separate binary logistic regression analyses were used to explore variables predicting progression to HD and persistence of hoarding symptom at the end of follow-up. In the primary models, baseline hoarding symptom severity and gender were included based on our hypothesis developed considering prior literature. In exploratory models, current psychiatric comorbidities and history of any psychiatric treatment during the follow-up period were entered to examine their potential confounding effects to progression and persistence outcomes. Current psychiatric comorbidity was defined as the presence of any psychiatric diagnosis identified at the four-year follow-up assessment. These variables were added separately to the primary model to avoid model over-specification and to assess whether associations observed in the primary analyses remained stable.

Model assumptions were evaluated prior to interpretation. Linearity of the logit for continuous predictors was assessed using the Box–Tidwell procedure. Multicollinearity was examined using variance inflation factors (VIF). Model fit was evaluated using the likelihood ratio (LR) chi-square statistic and Nagelkerke’s pseudo-R². Discriminative ability was quantified using the area under the receiver operating characteristic curve (AUC).

Given the modest sample size and limited number of outcome events in some analyses, Firth’s bias-reduced penalized logistic regression was performed as a sensitivity analysis using R statistical software (R Foundation for Statistical Computing, Vienna, Austria) with the logistf package to reduce potential small-sample bias and rare-event inflation of maximum likelihood estimates [17]. Penalized results were compared with binary logistic regression estimates to evaluate the robustness and stability of findings. Statistical significance was set at *p* < 0.05 (two-tailed).

## Results

### Longitudinal course of hoarding behavior

Among the participants followed up in the present study (*n* = 45), 42.2% (*n* = 19) progressed to HD, 20% (*n* = 9) continued to exhibit HB, and 37.8% (*n* = 17) no longer had hoarding symptoms. The four-year longitudinal course of HB is summarized in Fig. 1.

### Diagnostic progression to hoarding disorder

A statistically significant difference was found between the two groups in terms of gender (*p* = 0.009). Among adolescents who progressed to HD, 89.5% (*n* = 17) were female, whereas 50% (*n* = 13) of those who did not progress to HD were female. Compared to adolescents who did not progress to HD (30.8%, *n* = 8), those who progressed to HD (63.2%, *n* = 12) had experienced significantly more stressful life events (*p* = 0.031). No statistically significant differences were found between these two groups in terms of age, family history of psychiatric disorders, family income status, family type, and history of any psychiatric treatment. In addition, 57.9% (*n* = 11) of adolescents who progressed to HD also exhibited a comorbid diagnosis. Importantly, no participant had a clinically identified psychiatric diagnosis at baseline; thus, comorbid diagnoses observed at follow-up reflect incident psychopathology emerging during the longitudinal course. The most common comorbid diagnoses were anxiety disorder (42.1%, *n* = 8) and ADHD (26.3%, *n* = 5). A significant difference was found between the two groups in terms of any psychiatric disorder, anxiety disorder and ADHD comorbidities, and these comorbidities were found to be higher in the progressed to HD (*p* = 0.003, *p* = 0.006, *p* = 0.029 respectively). Other comorbid diagnoses observed adolescents progressed to HD were found to be depression (10.5%, *n* = 2), OCD (10.5%, *n* = 2), oppositional defiant disorder (ODD) (5.3%, *n* = 1), disruptive mood dysregulation disorder (DMDD) (5.3%, *n* = 1) and internet gaming disorder (IGD) (5.3%, *n* = 1), respectively. No statistically significant differences were found between two groups in terms of these disorders. Significant differences in hoarding symptoms severity were found both at baseline (57.11 vs. 46.38) and current (64.42 vs. 20.81), and were higher in adolescents who progressed to HD (*p* < 0.001). The comparison of demographic and clinical characteristics of participants who progressed to HD (*n* = 19) and who did not progress HD (*n* = 26) at the end of follow-up is shown in Table 1.

**Table 1:** Comparison of demographic and clinical characteristics of participants with and without progression to hoarding disorder outcome at follow-up

Variable	Progressed HD (*n* = 19)	Did not progress HD (*n* = 26)	Statistic	*P* value
Age (years)				
Mean ± SD	16.32 ± 1.29	16.73 ± 1.43	0.208^a^	0.323
Gender, n (%)				
Female Male	17 (89.5) 2 (10.5)	13 (50) 13 (50)	FET	**0.009**
Family history of psychiatric disorder, n (%)	5 (26.3)	9 (34.6)	0.353^b^	0.553
Family income, n (%)				
Low Middle High	0 (0) 17 (89.5) 2 (10.5)	6 (23.1) 18 (69.2) 2 (7.7)	FET	0.077
Family type, n (%)				
Nuclear family Extended family Fragmented family	16 (84.2) 2 (10.5) 1 (5.3)	20 (76.9) 6 (23.1) 0 (0)	FET	0.299
Stressful life events, n (%)	12 (63.2)	8 (30.8)	4.664^b^	**0.031**
History of any psychiatric treatment, n (%)	7 (36.8)	5 (19.2)	1.741^b^	0.187
Current Psychiatric Comorbidities, n (%)				
Any disorder Anxiety disorder ADHD Depression OCD ODD DMDD IGD	11 (57.9) 8 (42.1) 5 (26.3) 2 (10.5) 2 (10.5) 1 (5.3) 1 (5.3) 1 (5.3)	4 (15.4) 2 (7.7) 1 (3.8) 1 (3.8) 0 (0) 1 (3.8) 1 (3.8) 0 (0)	FET FET FET FET NA FET FET NA	**0.003** **0.006** **0.029** 0.565 NA 0.821 0.821 NA
Hoarding symptoms severity				
Baseline CSI, Mean ± SD	57.11 ± 5.33	46.38 ± 4.35	2.474^a^	**< 0.001**
Current CSI, Mean ± SD	64.42 ± 5.48	20.81 ± 10.78	-5.680^c^	**< 0.001**

Bold p values indicate values with significance at *p* < 0.05

^a^Independent samples t-test

^b^Pearson’s chi-squared test

^c^Mann Whitney U test

FET fischer’s exact test; HD hoarding disorder; ADHD attention-deficit/hyperactivity disorder; OCD obsessive–compulsive disorder; ODD oppositional defiant disorder; DMDD disruptive mood dysregulation disorder; IGD internet gaming disorder; CSI children’s saving inventory

### Persistence of hoarding symptoms

The comparison of demographic and clinical characteristics of participants with persistence of hoarding symptoms (*n* = 28) and absence of hoarding symptoms (*n* = 17) at the end of follow-up is shown in Table 2. Persistence of hoarding symptoms group consists of HD and HB. Compared to adolescents without hoarding symptoms (41.2%, *n* = 7), female sex was found to be significantly higher in adolescents with persistent hoarding symptoms (82.1%, *n* = 23) (*p* = 0.005). Statistically significant differences were found between the two groups in terms of stressful life events (57.1% vs. 23.5%), any disorder (50% vs. 5.9%), baseline (53.75 vs. 46.24) and current (52.04 vs. 18.12) hoarding symptoms severity and these variables were higher in adolescents with persistent hoarding symptoms (*p* = 0.035, *p* = 0.003, *p* < 0.001 respectively). On the other hand, no statistically significant differences were found between these two groups in terms of age, family history of psychiatric disorder, family income status, family type and history of any psychiatric treatment.

**Table 2:** Comparison of demographic and clinical characteristics of participants with persistence of hoarding symptoms and absence of hoarding symptoms outcome at follow-up

Variable	Persistence of hoarding symptoms (*n* = 28)	Absence of hoarding symptoms (*n* = 17)	Statistic	*P* value
Age (years)				
Mean ± SD	16.43 ± 1.32	16.76 ± 1.48	0.254^a^	0.433
Gender, n (%)				
Female Male	23 (82.1) 5 (17.9)	7 (41.2) 10 (58.8)	7.988^b^	**0.005**
Family history of psychiatric disorder, n (%)	8 (28.6)	6 (35.3)	0.353^b^	0.637
Family income, n (%)				
Low Middle High	1 (3.6) 24 (85.7) 3 (10.7)	5 (29.4) 11 (64.7) 1 (5.9)	FET	0.061
Family type, n (%)				
Nuclear family Extended family Fragmented family	21 (75) 6 (21.4) 1 (3.6)	15 (88.2) 2 (11.8) 0 (0)	FET	0.661
Stressful life events, n (%)	16 (57.1)	4 (23.5)	FET	**0.035**
History of any psychiatric treatment, n (%)	9 (32.1)	3 (17.6)	FET	0.488
Current Psychiatric Comorbidities, n (%)				
Any disorder Anxiety disorder ADHD Depression OCD ODD DMDD IGD	14 (50) 9 (32.1) 6 (21.4) 3 (10.7) 2 (7.1) 2 (7.1) 2 (7.1) 1 (3.6)	1 (5.9) 1 (5.9) 0 (0) 0 (0) 0 (0) 0 (0) 0 (0) 0 (0)	FET FET NA NA NA NA NA NA	**0.003** 0.064 NA NA NA NA NA NA
Hoarding symptoms severity				
Baseline CSI, Mean ± SD	53.75 ± 7.01	46.24 ± 4.49	7.677^a^	**< 0.001**
Current CSI, Mean ± SD	52.04 ± 19.98	18.12 ± 9.29	-4.510^c^	**< 0.001**

Persistence of hoarding symptoms group consists of HD and HB

Bold p values indicate values with significance at *p* < 0.05

^a^Independent samples t-test

^b^Pearson’s chi-squared test

^c^Mann Whitney U test

FET fischer’s exact test; HD hoarding disorder; HB hoarding behavior; ADHD attention-deficit/hyperactivity disorder; OCD obsessive–compulsive disorder; ODD oppositional defiant disorder; DMDD disruptive mood dysregulation disorder; IGD internet gaming disorder; CSI children’s saving inventory

### Predictors of outcome

We performed two separate binary logistic regression analyses to investigate possible predictors of diagnostic progression to HD and persistence of hoarding symptoms at four-year follow-up. In the primary models, gender and baseline hoarding symptoms severity were included. For progression to HD, the primary model was statistically significant (Model χ²(2) = 33.43, *p* < 0.001; Nagelkerke R² = 0.705; AUC = 0.94; overall classification accuracy = 88.9%). Baseline hoarding symptom severity significantly predicted diagnostic progression (OR = 1.42, *p* = 0.001), whereas gender was not a significant predictor. For persistence of hoarding symptoms, the model was also statistically significant (Model χ²(2) = 17.28, *p* < 0.001; Nagelkerke R² = 0.434; AUC = 0.83; overall classification accuracy = 77.8%). Baseline hoarding symptom severity was associated with persistence (OR = 1.20, *p* = 0.011), whereas gender was not a significant predictor. The results of the primary models are presented in Table 3.

**Table 3:** Logistic regression analysis examining predictors of progression to HD and persistence of hoarding symptom

	B	SE	*p*	OR	95% CI
Progression to HD^a^					
Gender^c^	1.484	1.191	0.213	4.41	0.43–45.54
Baseline hoarding symptoms severity^d^	0.350	0.109	**0.001**	1.42	1.15–1.76
Persistence of hoarding symptoms^b^					
Gender^c^	1.374	0.780	0.078	3.95	0.86–18.22
Baseline hoarding symptoms severity^d^	0.186	0.074	**0.011**	1.20	1.04–1.44

Persistence of hoarding symptoms group consists of HD and HB

Bold p values indicate values with significance at *p* < 0.05

^a^Model χ2 = 33.43, Prob > χ2 = < 0.001, Nagelkerke pseudo R² = 0.705, Classification Accuracy = 88.9%, AUC = 0.94

^b^Model χ2 = 17.28, Prob > χ2 = < 0.001, Nagelkerke pseudo R² = 0.434, Classification Accuracy = 77.8%, AUC = 0.83

^c^Female

^d^Children’s Saving Inventory

HD hoarding disorder; HB hoarding behavior

In exploratory models, current psychiatric comorbidities (defined as the presence of any psychiatric disorder at follow-up) and history of any psychiatric treatment during the follow-up period were entered separately into the primary models to evaluate their potential contribution to progression and persistence outcomes. When current psychiatric comorbidity was added to the progression model, baseline hoarding symptom severity remained a significant predictor of progression to HD (OR = 1.39, *p* = 0.006), whereas gender and comorbidity were not independently associated with progression. In contrast, in the persistence model, both female gender (OR = 14.36, *p* = 0.026) and current psychiatric comorbidities (OR = 31.36, *p* = 0.011) were significantly associated with persistence of hoarding symptoms, while baseline hoarding severity also remained significant (OR = 1.22, *p* = 0.040). The results are reported in Supplementary Table S1.

When history of any psychiatric treatment was added to the progression model, baseline hoarding symptom severity remained the only significant predictor (OR = 1.40, *p* = 0.001), whereas gender and treatment history were not significantly associated with progression to HD. Similarly, in the persistence model including treatment history, baseline hoarding symptoms severity remained significantly associated with persistence (OR = 1.19, *p* = 0.013), while neither gender nor treatment history independently predicted persistence. Importantly, treatment history during follow-up was not independently associated with progression to HD or persistence of hoarding symptoms in exploratory analyses. These findings are presented in Supplementary Table S2.

Sensitivity analyses using Firth’s bias-reduced penalized logistic regression yielded directionally consistent findings across all models. The pattern of statistical significance did not change compared to binary logistic regression analyses. Although effect size estimates were slightly attenuated under penalized estimation in some models, these findings support the robustness of the observed associations. Detailed results are provided in Supplementary Table S3.

For all logistic regression models, assumption checks indicated that the linearity of the logit was satisfied (Box–Tidwell interaction terms, all *p* > 0.05) and no evidence of multicollinearity was detected (all VIF values < 2).

## Discussion

To our knowledge, this is the first follow-up study of HB, highlighting the clinical significance of the unique outcomes regarding the longitudinal course of HB and its progression to HD. To clarify the course and identify predictors of outcome for the first time, we prospectively evaluated children and adolescents with HB in our previous study and conducted detailed psychiatric interviews four years after the initial assessment. Across a four-year period, adolescents exhibited heterogeneous trajectories, including diagnostic progression to HD (42.2%), persistence of HB (20%), and absence of hoarding symptoms (37.8%). Regarding outcome predictors, baseline hoarding symptom severity significantly predicted both diagnostic progression and persistence across primary models. In exploratory analyses, baseline severity remained the only independent predictor of progression to HD, whereas persistence of hoarding symptoms was additionally associated with female gender and current psychiatric comorbidity. History of any psychiatric treatment during follow-up was not independently associated with either progression or persistence outcomes.

Studies of hoarding in childhood and adolescence have predominantly focused on symptoms or behaviors rather than the disorder itself. It is well known that disorders diagnosed through clinical interviews cannot be categorized in the same way as conditions with symptomatic manifestations [18]. Symptom presence alone does not necessarily correspond to a diagnosable disorder, particularly in developmental contexts where environmental and parental factors influence symptom expression [19]. In fact, results from our previous study indicate that HD and HB exhibit clinically significant differences, and therefore, the conditions labeled as behaviors cannot necessarily be considered disorders [4]. The distinction between HB and HD remains a matter of debate. Most existing studies have identified HB using screening instruments rather than clinical diagnostic interviews. To date, only one study – our previous study - has clinically evaluated HB by endorsing DSM-5 criteria [4]. In that framework, HD was diagnosed when all DSM-5 criteria were met, whereas HB was defined as the presence of criteria A and B in the absence of criterion C (accumulation and clutter) and criterion D (clinically significant distress or impairment of functioning). By contrast, one of the few clinical studies have adopted broader definitions, classifying individuals who met only criteria A and B within the HD (hoarding group) [10]. Given the limited autonomy of children and adolescents due to parental intervention, the validity of the clutter criterion (criterion C) remains debated. Developmental considerations are often necessary when evaluating clutter severity. In the present study, diagnostic determinations were grounded in strict adherence to criterion D while incorporating developmental considerations when assessing criterion C. This approach ensured alignment with DSM standards while accounting for the unique contextual features of adolescence.

Notably, almost half (42.2%) of participants with HB progressed to HD over a four-year period, indicating that hoarding symptoms in adolescence are not merely transient developmental phenomena. Although prior longitudinal research has reported comparable rates of persistent hoarding symptoms [10], methodological differences complicate direct comparison. The previous study relied on screening instruments and did not require endorsement of DSM-5 criteria C and D, whereas the present study applied clinical interviews and required clinically significant impairment for diagnosis. Differences in baseline age and follow-up duration may also have influenced observed trajectories. Although there are methodological differences between these two longitudinal studies conducted in childhood and adolescence, a significant proportion of participants with hoarding symptoms in the past also exhibited hoarding symptoms at follow-up. Beyond methodological variation, these findings support the notion that hoarding symptoms emerging in early adolescence may represent the early manifestation of a persistent vulnerability rather than a temporary behavioral pattern. Over time, hoarding behaviors may consolidate into a disorder that leads to clinical impairment. This developmental progression highlights the importance of identifying youth at risk before diagnosable disorder.

In the present study, children and adolescents with HB demonstrated a substantial risk of progression to HD within a four-year period. However, childhood and adolescence represent relatively limited developmental windows, and longitudinal studies conducted within this age range necessarily capture only a segment of the disorder’s potential trajectory. Evidence from adult retrospective studies indicates that hoarding symptoms often emerge during adolescence, whereas a diagnosis of HD is more frequently established in early adulthood, suggesting a temporal gap between initial symptom onset and diagnostic threshold crossing [6, 20, 21]. These findings imply that the four-year follow-up period may capture only the early phase of a longer developmental process. In light of findings from these three retrospective adult studies, it is reasonable to hypothesize that a greater number of participants with HB would have progressed to HD if the study had been conducted over a longer period. This highlights the need for longer prospective studies spanning the transition from adolescence into adulthood to more fully delineate the course of hoarding.

Participants who progressed to HD and those who did not exhibited clinically significant differences. First, female gender dominance was evident among adolescents who progressed to HD. This finding aligns with previous studies reporting that hoarding symptoms are significantly more prevalent females than in males [4, 10, 22]. A notable feature of adolescents who progressed to HD was their increased likelihood of experiencing stressful life events. This is consistent with findings showing that hoarding symptoms are highly associated with stressful life events in both childhood and adulthood [4, 20, 21]. However, the temporal and causal relationship remains uncertain in this age group. Stressors may contribute to escalation of saving and acquisition as maladaptive coping strategies, but it is also plausible that emerging hoarding-related impairment increases interpersonal conflict and contextual stress over time. Prospective studies with more fine-grained assessment of timing and severity of stressors are needed to clarify these pathways.

A noteworthy finding was that adolescents who progressed to HD were also more likely to exhibit comorbid diagnoses. The fact that more than half of the adolescents with HD had comorbid diagnoses supports findings from previous studies [4, 10]. The most common comorbid diagnoses were anxiety disorders and ADHD. The pattern of psychopathology observed was similar to that reported in the existing literature on children and adolescents with HD [4, 10]. Youths with hoarding symptoms exhibit high comorbidity with both internalizing and externalizing disorders [23]. Although the temporal sequence of these conditions cannot be definitively established, the co-occurrence of HD with anxiety and ADHD raises the possibility of shared vulnerability mechanisms. Emotional and behavioral dysregulation may represent transdiagnostic processes contributing to both hoarding pathology and comorbid internalizing or externalizing symptoms. The presence of diverse comorbid profiles further suggests that HB may follow heterogeneous developmental pathways rather than a single etiological trajectory.

Logistic regression analysis examining the predictors of progression to HD and persistence of hoarding symptoms provided a novel insight. Baseline hoarding symptom severity consistently predicted both progression to HD and persistence of hoarding symptoms across primary and exploratory models. Importantly, when current psychiatric comorbidity and treatment history were entered separately into the models, baseline severity remained the only independent predictor of progression to HD. In contrast, persistence of hoarding symptoms was predicted not only by baseline severity but also by female gender and current psychiatric comorbidity, whereas treatment exposure was not independently associated with outcomes. Since there are no longitudinal studies in the scientific literature examining predictors of progression to HD or hoarding symptom persistence in children and adolescents, there are no direct comparisons available. However, there are cross-sectional studies on this subject.

The finding that baseline hoarding symptom severity predicted progression to HD is consistent with adult literature demonstrating that hoarding severity is closely linked to clinical impairment and chronicity [24–26]. Greater baseline severity may reflect more entrenched maladaptive decision-making patterns, stronger emotional attachment to possessions, and more pronounced executive dysfunction, thereby increasing the likelihood of crossing the diagnostic threshold. Emotional and cognitive factors such as anxiety sensitivity, impulsivity, and reduced distress tolerance have been shown to correlate with hoarding severity [27], and may represent underlying vulnerability processes contributing to progression. It is likely that the development of HD is the result of a complex interplay of these factors, but the exact nature of this interaction is not yet clear. Although not evaluated in the present study, these factors may have contributed to both baseline and current symptom severity and thus progression to HD.

The association between female gender and persistence of hoarding symptoms aligns with previous cross-sectional findings in youth samples [4]. Although the mechanisms remain uncertain, gender differences in emotional processing, internalizing symptom profiles, or help-seeking patterns may contribute to sustained symptom expression. Another possibility is that hoarding, anxiety and ADHD share common symptoms. In anxious children, attention problems have been found to predict hoarding symptomatology beyond the contribution of anxiety symptoms [9]. A study conducted in adults showed that ADHD symptoms (especially inattention) are a key predictor of hoarding-related behaviors [28]. In another adult study, anxiety symptoms were found to be a significant predictor of hoarding symptoms [29]. Moreover, the independent contribution of psychiatric comorbidity to persistence suggests that co-occurring internalizing and externalizing disorders may function as maintenance factors rather than drivers of diagnostic transition.

Taken together, the findings suggest that baseline hoarding severity functions as a core vulnerability marker influencing both diagnostic progression and persistence of symptoms. Rather than representing a factor limited to threshold crossing, severity appears to reflect the underlying intensity and consolidation of hoarding-related cognitive and behavioral patterns. In contrast, female gender and current psychiatric comorbidity may not independently drive progression to HD, but may contribute to the maintenance and stabilization of hoarding behaviors over time. Thus, while severity may represent the foundational risk dimension, comorbid psychopathology and gender-related factors may act as modifiers that influence the longitudinal expression and chronicity of hoarding symptoms. The interpretation of these findings should also consider potential unmeasured environmental and intervening factors. Academic pressure, family dynamics, changes in living conditions, and broader psychosocial stressors during adolescence may have influenced both symptom persistence and diagnostic progression. Although stressful life events were examined in bivariate analyses, more granular information regarding the timing, chronicity, and severity of environmental stressors was not available. In addition, although no participant received treatment specifically targeting hoarding symptoms, some adolescents were exposed to pharmacological or psychotherapeutic interventions for comorbid conditions during the follow-up period. While treatment exposure did not independently predict outcomes in exploratory analyses, it remains possible that indirect effects—such as partial symptom improvement, improved emotional regulation, or enhanced executive functioning—may have influenced longitudinal trajectories. Future studies incorporating detailed assessments of environmental context, family functioning, and treatment intensity are needed to clarify how these factors interact with baseline vulnerability to shape the developmental course of hoarding pathology.

### Clinical implications

The present findings carry several implications for clinical practice. First, HB in childhood and adolescence should not be considered a transient developmental phenomenon. A substantial proportion of adolescents with HB progressed to HD over a four-year period, underscoring the importance of early identification and monitoring. Similar to research on other subthreshold psychiatric conditions [30, 31], HB appears to represent a clinically meaningful risk state rather than a harmless behavioral variation. Second, baseline hoarding severity consistently predicted both diagnostic progression and symptom persistence, suggesting that dimensional assessment of symptom intensity may help clinicians identify youth at elevated longitudinal risk. Third, the association between psychiatric comorbidity and persistence of hoarding symptoms highlights the potential importance of transdiagnostic processes. Although comorbidity did not independently predict progression to HD, its relationship with persistence suggests that untreated internalizing or externalizing psychopathology may contribute to the stabilization of hoarding behaviors over time. From a clinical standpoint, this finding supports comprehensive assessment and evidence-based treatment of psychiatric conditions—particularly anxiety disorders and ADHD—as part of an integrated care approach. Furthermore, screening may be particularly relevant in clinical contexts where transdiagnostic vulnerabilities are common.

### Limitations

Although our study is the first in the literature in several respects, our results should be interpreted carefully, considering its limitations. First, the relatively small sample size limits statistical power and the precision of effect estimates. While the use of clinically confirmed DSM-5–based assessments enhanced diagnostic validity and reduced the likelihood of false-positive classification, it necessarily resulted in a more restricted sample. The modest number of outcome events may limit generalizability and increase uncertainty around parameter estimates, despite the robustness of findings across primary and penalized regression models. Second, the four-year follow-up period was relatively short, but a significant proportion of children and adolescents with HB progressed to HD. This finding challenges the notion that HD develops gradually over several decades. Third, although participants were reassessed after four years, detailed time-sequenced information regarding intervening experiences during the follow-up interval was not systematically collected. While none of the adolescents received treatment specifically targeting hoarding symptoms, some received pharmacological or psychotherapeutic interventions for psychiatric conditions. In addition, potential unmeasured factors such as academic stress, family dynamics, or unrecorded adverse life events may have affected symptom trajectories. Finally, certain variables were assessed using methods that introduce measurement limitations. Stressful life events during the follow-up period were evaluated retrospectively, which may be vulnerable to recall bias and does not capture timing, chronicity, or severity of exposure. Furthermore, although Criterion C was assessed with developmental flexibility to account for parental control of the living environment, this approach relies partly on parent report and clinical judgment rather than solely on observable clutter.

## Conclusion

To our knowledge, this is the first follow-up study of HB to utilize clinical interviews based on DSM-5 diagnostic criteria at both baseline and follow-up. Our findings offer valuable insights into the longitudinal course of HB and the factors influencing its progression. A substantial proportion of children and adolescents with HB progressed to HD over four years, highlighting that clinically significant hoarding behaviors may represent an early stage of a disorder rather than a transient developmental phenomenon. Baseline hoarding symptom severity emerged as the most consistent predictor of diagnostic progression to HD and was also associated with persistence of hoarding symptoms. In contrast, female gender and current psychiatric comorbidity were particularly relevant for symptom persistence, suggesting that comorbid psychopathology may contribute to the maintenance of hoarding symptoms over time. These findings underscore the importance of early identification of children and adolescent with HB and careful monitoring of those with co-occurring psychiatric conditions. Larger, multi-center longitudinal studies with repeated interim assessments are needed to further clarify developmental trajectories and to refine risk-based intervention strategies aimed at preventing progression to HD.

## Supplementary Information

Below is the link to the electronic supplementary material.

Supplementary Material 1

Supplementary Material 2

## Data Availability

The data that support the findings of this study are available from the corresponding author, upon reasonable request.

## Abbreviations

HB: Hoarding behavior
HD: Hoarding disorder
K-SADS-PL: Schedule for affective disorders and schizophrenia for school-age children-present and lifetime version
CSI: Children’s saving inventory
DSM-5: Diagnostic and statistical manual of mental disorders, fifth edition
OCD: Obsessive-compulsive disorder
ADHD: Attention deficit/hyperactivity disorder
ODD: Oppositional defiant disorder
DMDD: Disruptive mood dysregulation disorder
IGD: Internet gaming disorder
