# Supplementary material for: Diagnostic progression to hoarding disorder: the longitudinal course of hoarding behavior
Source: BMC Psychiatry. 2026 Apr 16;26:423. doi: 10.1186/s12888-026-08055-4 (PMC13202944; doi:10.1186/s12888-026-08055-4)
Supplement: Supplementary file 1 — Supplementary Material 1 [file 12888_2026_8055_MOESM1_ESM.docx]

| **Table S1 Logistic regression analysis examining predictors of progression to HD and persistence of hoarding symptom including current psychiatric comorbidities as covariates** | | | |
| --- | --- | --- | --- |
| Progression to HD^a^ | | | |
|  | *B* (*SE*) | *p* | OR [95% CI] |
| Gender^c^ | 1.748 (1.358) | .198 | 5.74 [0.40, 82.19] |
| Baseline hoarding symptoms severity^d^ | .330 (.120) | **.006** | 1.39 [1.10, 1.76] |
| Current Psychiatric Comorbidities | 2.211 (1.305) | .090 | 9.13 [0.71, 117.86] |
| Persistence of hoarding symptoms^b^ | | | |
|  | *B* (*SE*) | *p* | OR [95% CI] |
| Gender^c^ | 2.665 (1.194) | **.026** | 14.36 [1.38, 149.09] |
| Baseline hoarding symptoms severity^d^ | .202 (.098) | **.040** | 1.22 [1.01, 1.48] |
| Current Psychiatric Comorbidities | 4.268 (1.670) | **.011** | 31.36 [2.71, 356.85] |
| *Persistence of hoarding symptoms group consists of HD and HB*  *Bold p values indicate values with significance at p < 0.05*  *^a^Model χ2 = 36.86, Prob > χ2 = <0.001, Nagelkerke pseudo R² = 0.752, Classification Accuracy = 86.7%, AUC = 0.96.*  *^b^Model χ2 = 28.23, Prob > χ2 = <0.001, Nagelkerke pseudo R² = 0.635, Classification Accuracy = 84.4%, AUC = 0.92.*  *^c^Female*  *^d^Children's saving inventory*  *HD hoarding disorder*  *HB hoarding behavior* | | | |
| **Table S2 Logistic regression analysis examining predictors of progression to HD and persistence of hoarding symptom including** **any psychiatric treatment as covariates** | | | |
| Progression to HD^a^ | | | |
|  | *B* (*SE*) | *p* | OR [95% CI] |
| Gender^c^ | 1.503 (1.202) | .211 | 4.49 [0.43, 47.39] |
| Baseline hoarding symptoms severity^d^ | .335 (.106) | **.001** | 1.40 [1.14, 1.72] |
| Any Psychiatric Treatment | .814 (1.086) | .453 | 2.26 [0.27, 18.96] |
| Persistence of hoarding symptoms^b^ | | | |
|  | *B* (*SE*) | *p* | OR [95% CI] |
| Gender^c^ | 1.488 (.807) | .065 | 4.43 [0.91, 21.53] |
| Baseline hoarding symptoms severity^d^ | .177 (.072) | **.013** | 1.19 [1.04, 1.37] |
| Any Psychiatric Treatment | .949 (1.004) | .344 | 2.58 [0.36, 18.47] |
| *Persistence of hoarding symptoms group consists of HD and HB*  *Bold p values indicate values with significance at p < 0.05*  *^a^Model χ2 = 33.99, Prob > χ2 = <0.001, Nagelkerke pseudo R² = 0.713, Classification Accuracy = 82.2%, AUC = 0.95.*  *^b^Model χ2 = 18.22, Prob > χ2 = <0.001, Nagelkerke pseudo R² = 0.453, Classification Accuracy = 77.8%, AUC = 0.84.*  *^c^Female*  *^d^Children's saving inventory*  *HD hoarding disorder*  *HB hoarding behavior* | | | |
