# Supplementary material for: Diagnostic progression to hoarding disorder: the longitudinal course of hoarding behavior
Source: BMC Psychiatry. 2026 Apr 16;26:423. doi: 10.1186/s12888-026-08055-4 (PMC13202944; doi:10.1186/s12888-026-08055-4)
Supplement: Supplementary file 2 — Supplementary Material 2 [file 12888_2026_8055_MOESM2_ESM.docx]

**Table S3. Firth’s Bias-Reduced Logistic Regression Predicting Progression to Hoarding Disorder and Persistence of Hoarding Symptoms**

|  | **Progression to HD** | | | **Persistence of Hoarding Symptoms** | | |
| --- | --- | --- | --- | --- | --- | --- |
|  | Model 1 | Model 2 | Model 3 | Model 1 | Model 2 | Model 3 |
| Gender^a^ | 3.50 (0.43–28.22) | 3.73 (0.40–34.74) | 3.41 (0.43–26.90) | 3.58 (0.81–15.94) | 8.74 (1.16–65.74)* | 3.78 (0.83–17.20) |
| Baseline hoarding symptoms severity^b^ | 1.34 (1.13–1.60)*** | 1.30 (1.08–1.57)** | 1.31 (1.11–1.55)*** | 1.18 (1.03–1.34)* | 1.17 (1.00–1.38)* | 1.16 (1.02–1.32)* |
| Current Psychiatric Comorbidities | — | 5.82 (0.67–50.57) | — | — | 21.97 (1.82–230.06)* | — |
| Any Psychiatric Treatment | — | — | 2.14 (0.31–14.88) | — | — | 2.27 (0.36–14.09) |

Model 1 included gender and baseline hoarding severity

Model 2 included gender, baseline hoarding symptoms severity and current psychiatric comorbidities

Model 3 included gender, baseline hoarding symptoms severity and any psychiatric treatment

^a^Female

^b^Children's Saving Inventory

Persistence of hoarding symptoms group consists of HD and HB

HD hoarding disorder

HB hoarding behavior

Values represent odds ratios (95% confidence intervals) estimated using Firth’s bias-reduced penalized logistic regression.

* p < .05; ** p < .01; *** p < .001.
